# Sperm Microbiota and Its Impact on Semen Parameters

**DOI:** 10.3389/fmicb.2019.00234

**Published:** 2019-02-12

**Authors:** David Baud, Céline Pattaroni, Nicolas Vulliemoz, Vincent Castella, Benjamin J. Marsland, Milos Stojanov

**Affiliations:** ^1^Materno-fetal and Obstetrics Research Unit, Department Woman Mother Child, Lausanne University Hospital, Lausanne, Switzerland; ^2^Department of Immunology and Pathology, Monash University, Melbourne, VIC, Australia; ^3^Service de Pneumologie, Lausanne University Hospital, Lausanne, Switzerland; ^4^Fertility Medicine and Gynaecologic Endocrinology Unit, Department Woman Mother Child, Lausanne University Hospital, Lausanne, Switzerland; ^5^Forensic Genetics Unit, University Center of Legal Medicine Lausanne-Geneva, Lausanne, Switzerland

**Keywords:** microbiota, spermatozoa, infertility, Prevotella, *Lactobacillus*

## Abstract

Compared to its female counterpart, the microbiota of the male genital tract has not been studied extensively. With this study, we aimed to evaluate the bacterial composition of seminal fluid and its impact on sperm parameters. We hypothesized that a dysbiotic microbiota composition may have an influence on sperm quality. Semen samples of 26 men with normal spermiogram and 68 men with at least one abnormal spermiogram parameter were included in the study. Samples were stratified based on total sperm count, spermatozoa concentration, progressive motility, total motility and spermatozoa morphology. Microbiota profiling was performed using 16S rRNA gene amplicons sequencing and total bacterial load was determined using a panbacterial quantitative PCR. Semen samples broadly clustered into three microbiota profiles: *Prevotella*-enriched, *Lactobacillus*-enriched, and polymicrobial. *Prevotella*-enriched samples had the highest bacterial load (*p* < 0.05). Network analysis identified three main co-occurrence modules, among which two contained bacteria commonly found in the vaginal flora. Genera from the same module displayed similar oxygen requirements, arguing for the presence of different ecological niches for bacteria that colonize semen through the passage. Contrary to our hypothesis, shifts in overall microbiota composition (beta-diversity) did not correlate with spermiogram parameters. Similarly, we did not find any difference in microbial richness or diversity (alpha-diversity). Differential abundance testing, however, revealed three specific genera that were significantly enriched or depleted in some of the sperm quality groups (*p* < 0.05). *Prevotella* relative abundance was increased in samples with defective sperm motility while *Staphylococcus* was increased in the corresponding control group. In addition, we observed an increased relative abundance of *Lactobacillus* in samples with normal sperm morphology. Our study indicates that overall bacterial content of sperm might not play a major role in male infertility. Although no major shifts in microbiota composition or diversity were found, the differential abundance of specific bacterial genera in the sperm suggests that a small subset of microbes might impact the spermatozoal physiology during sperm transition, more specifically motility and morphology. Further studies are required to challenge this finding and develop potential strategies to induce the formation of a healthy seminal microbiota.

## Introduction

In several circumstances, male infertility has been linked to bacterial infections of the genital tract (Gimenes et al., 2014), which might cause inflammation of tissues, obstruction of genital ducts, epididymitis and orchitis among others. Moreover, bacteria may have a direct negative impact on spermatozoa physiology, reducing viability or motility (Reichart et al., 2000; Hosseinzadeh et al., 2001; Baud et al., 2017) but the true impact of bacterial infections on male fertility remains controversial. Although bacteriospermia was previously considered to be negatively associated with fertility, recent studies indicate that presence of bacteria in semen is relatively frequent, including in fertile individuals with normal sperm parameters (Cottell et al., 2000; Rodin et al., 2003). As with other sites of the human body, it appears that semen has a specific microbiota and it can be postulated that presence of a specific bacterial milieu may not be deleterious but necessary for normal sperm function (Hou et al., 2013; Weng et al., 2014; Mändar et al., 2015).

Compared to other body sites, the seminal microbiota has been minimally investigated. Initial studies based on culture-dependent methods, targeted PCR amplification of ribosomal RNA gene sequences and microscopy, underestimated the abundance of bacteria in semen and focused mainly on the detection of known pathogens. With the advent of next generation sequencing it became possible to elucidate the bacterial composition of semen with higher accuracy and gain more insight into its interaction with the host. The host immune system may play a crucial role in the dynamics of the semen microbiota, since its activation during infections is related to significant changes in the microbiota composition. HIV infection was associated with decreased semen microbiota diversity and richness (Liu et al., 2014), while higher species diversity in semen was observed in patients with prostatitis compared to control group, in addition to a reduction in the relative abundance of lactobacilli (Mändar et al., 2017).

In male infertility, it would be important to evaluate whether specific microbiological signatures correlate with the fertility status of the individual. In an initial study, Hou et al. (2013) identified six microbiota clusters, none of which was specifically associated with infertility. The presence of *Anaerococcus* in semen, however, was negatively associated with its quality. A second study clustered bacterial content of semen into three groups, two of which, *Pseudomonas*- and *Prevotella*-predominant, were associated with abnormal semen parameters (Weng et al., 2014). In the third group, *Lactobacillus*-predominant, a higher proportion of normospermic patients was observed.

The dearth of studies warrants further research on the impact of the seminal microbiome on male fertility and infertility. In this work, we describe the bacterial composition of semen in 94 patients from infertile couples and its association with male fertility. Based on spermiogram analysis, we divided the subjects into normospermic and abnormal sperm parameter groups, in order to assess whether specific microbiota or bacteria are associated with abnormal semen parameters. The patients enrolled were mainly European and the seminal microbiota has not yet been explored in depth in this population, with the exception of a study including 20 individuals (Mändar et al., 2015). The two above mentioned studies were conducted on patients of Asian origin (China and Taiwan, respectively), but due to the geographical variation of the microbiota seen in different body sites (Suzuki and Worobey, 2014; Gupta et al., 2017) and the discordant conclusions, it is essential to assess the impact of seminal microbiota on semen function in a different population.

## Materials and Methods

### Semen Samples

Samples analyzed in this study were obtained from the Lausanne University Hospital Fertility Medicine Unit between October 2014 and July 2016. This study was carried out in accordance with the recommendations of the Cantonal Human Research Ethics Commission of Vaud (CER-VD), according to the Swiss Federal Act on Research involving Human Beings. The protocol was approved by the CER-VD (protocol 265-14). All patients were fully informed of the research project and gave their written consent to participate in the study. Samples were processed for routine semen assessment at the Laboratory of andrology and reproductive biology (LABR), according to WHO guidelines (World Health Organization, 2010). Semen was collected after 2 to 5 days of sexual abstinence by masturbation and examined following 30 min liquefaction at 37°C. The samples were manually evaluated for volume and pH, and then assessed using optical microscopy for concentration and morphology. Concentration and motility (total and progressive) evaluation were performed using the computer-assisted sperm analysis tool, CASA SCA (5.4, Microptic SL, Barcelona, Spain). For morphology, smears were stained using the Papanicolaou method and examined under a microscope at 100× magnification.

### DNA Extraction and Library Preparation

DNA extraction from total ejaculates was performed as previously described (Baud et al., 2017). In addition, two extraction negative controls, in which sterile H_2_O was processed the same way as the samples, were included in the study. Briefly, QIAamp DNA mini kit (Qiagen AG, Basel, Switzerland) was used following the manufacturer’s protocol, with the addition of 43 mM DTT to the lysis buffer. Bacterial DNA was amplified using custom barcoded primers targeting the V1-V2 region of the 16S rRNA gene (F-27/R-338) with Illumina sequencing adaptors, as previously described (Rapin et al., 2017). Each sample was amplified using the Kapa HiFi PCR Kit (KAPA Biosystems, Cape Town, South Africa). Cycling conditions consisted of 3 min of denaturation at 95°C, 30 cycles of 30 s at 98°C, 30 s at 56°C and 1 min 30 s at 72°C, ended by a final extension step of 5 min at 72°C. Amplicons were quantified using the LabChip GX instrument (Perkin Elmer, Waltham, MA, United States), pooled at equimolar amounts and purified using AMPure XP bead clean-up system (Beckman Coulter). The library was diluted to 12 pM and spiked with 25% phiX before loading into an Illumina MiSeq instrument using the v2-500 (paired-end, 2×250) reagent kit (Illumina, San Diego, CA, United Sates).

### Bacterial 16S rRNA Sequences Preprocessing

Raw sequences were processed using Quantitative Insights into Microbial Ecology (QIIME, v1.9.1) software (Caporaso et al., 2010). Paired forward and reverse sequencing reads were assembled using fastq-join, demultiplexed based on their nucleotide bar-code and quality filtered (quality Phred score Q < 20, more than 3 low-quality base calls, more than 75% of their original length). *De novo* chimera detection and removal were performed using usearch61 (Edgar, 2010). Demultiplexed sequences were merged to Operational Taxonomic Units (OTUs) with usearch61 at 97% identity threshold using a closed reference picking strategy against the 97% Greengenes reference database (v13.5) (DeSantis et al., 2006). 78.56% of the initial 6,540,482 quality and chimera filtered reads matched the reference database. Samples with less that 10,000 high-quality reads (which also included extraction negative controls) were excluded. Final OTU table was then normalized using a single rarefaction at 10,000 sequences depth. All downstream analyses were performed at genus-level using R statistical software. The sequences reported in this paper have been deposited in the National Center for Biotechnology Information Sequence Read Archive (BioProject accession number PRJNA509076).

### Clustering Into Microbiota Profiles and Diversity Testing

Alpha diversity was estimated using the chao1 and Shannon indexes calculated in QIIME. Beta diversity was visualized using Principal Coordinate Analysis (PCoA) on the Bray-Curtis dissimilarity distance matrix at the genus level. Samples were assigned to microbiota profiles using Partitioning Around Medoid clustering (PAM) algorithm. Analysis of similarity (ANOSIM) was performed with 999 random permutations on the same Bray Curtis distance matrix to test for differences in microbiota composition among the different groups.

### Co-occurrence Network Analysis and Differential Abundance Testing

Microbiota community structure was evaluated by building co-occurrence networks of the most abundant genera (> 1% mean relative abundance in the global dataset) using the Sparse Correlations for Compositional data (SparCC) algorithm (Friedman and Alm, 2012). Pseudo *p*-values were calculated using a bootstrap procedure with 999 random permutations and 999 iterations for each SparCC calculation. Significant associations were defined as positive SparCC correlations with a *p*-value < 0.05. An undirected network, weighted by SparCC correlation magnitude, was generated using the igraph package. Linear discriminant analysis (LDA) effect size (LEfSe) was used to identify differentially abundant bacterial genera among sperm parameters. Bacterial genera with a LDA score > 2 and adjusted *p*-value < 0.05 were considered significant (Segata et al., 2011).

### Quantification of Total Bacterial Load

Quantification of 16S rDNA copy numbers was performed using the method described by Castillo et al. (Castillo et al., 2006). We used the KAPA SYBR^®^ FAST Universal Kit (KAPA Biosystems), following manufacturer’s protocol and using 300 nM of both primers F-tot (5′-GCAGGCCTAACACATGCAAGTC-3′) and R-tot (5′-CTGCTGCCTCCCGTAGGAGT-3′).

### Statistical Analyses

Differences in bacterial load, richness, and diversity were evaluated using the non-parametric Kruskal–Wallis test followed by *post hoc* Wilcoxon rank sum test with continuity correction. Alpha level of significance was set to 0.05 for all statistical tests with *p*-value <0.05, <0.01, and <0.001 represented as ^∗^, ^∗∗^ and ^∗∗∗^, respectively.

## Results

### Study Population

**FIGURE 1:**
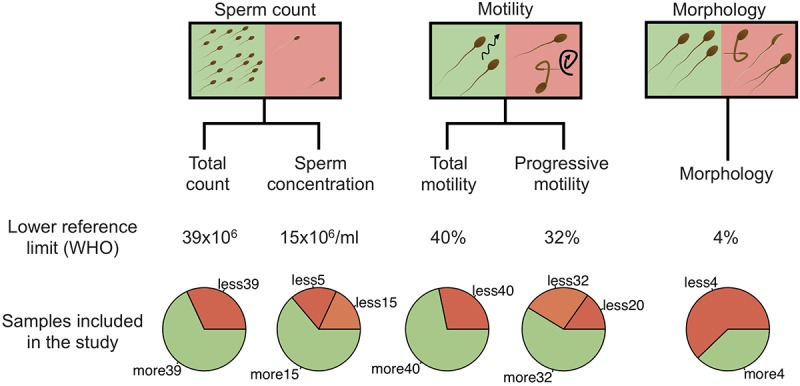
Schematic representation of sample stratification. In addition to the spermiogram status, five categories, including total sperm count, sperm concentration, total motility, progressive motility and morphology were analyzed independently by comparing normal and abnormal values. For sperm concentration and progressive motility categories, abnormal values were further separated into two classes (abnormal and severe). Lower reference limits, defined by the WHO, are shown for each category.

All men were generally in good health, without ongoing urogenital complications or sexual transmitted diseases. None was under antibiotic treatment at the time of the sampling. Among the 94 men included in the study, 26 had normal spermiogram parameters, while 68 had one or more abnormal parameters (total spermatozoa count, spermatozoa concentration, progressive spermatozoa motility, total spermatozoa motility, and spermatozoa morphology) according to the WHO guidelines (Supplementary Table 1). Schematic representation of sample stratification is depicted in Figure 1.

### Distinct Microbiota Profiles in Semen

**FIGURE 2:**
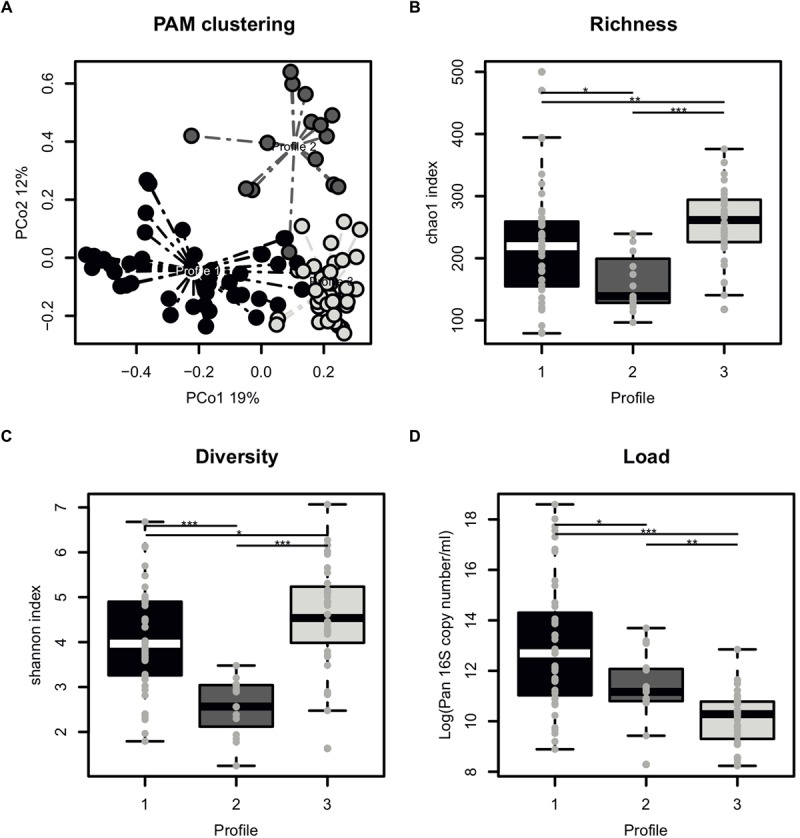
Characterization of semen microbiota profiles. **(A)** Principal Coordinate Analysis (PCoA) score plot on the Bray-Curtis distance at genus taxonomic level with each dot representing an individual patient explaining 19% (x axis) and 12% (y axis) of variance, respectively. Colors indicate microbiota profile, as defined by unbiased Partitioning Around Medoid clustering (PAM) clustering with dashed line representing connection to the cluster centroid. **(B)** Boxplot comparing the richness of microbiota profiles, as measured by chao1 index. **(C)** Boxplot comparing the diversity of microbiota profiles, as measured by chao1 index. **(D)** Boxplot comparing the total bacterial load of microbiota profiles, as determined by Pan 16S qPCR. Each dot represents an individual patient with mean boxplot indicating the mean plus or minus SD. Statistics represents the result of *post hoc* one-tailed Wilcoxon rank sum test.

The majority of samples had 10^4^–10^5^ 16S rRNA copies per ml of semen (Table 1). Overall, the most abundant bacterial genera in semen samples included members of Actinobacteria (*Corynebacterium*), Bacteroidetes (*Prevotella*), Firmicutes (*Lactobacillus*, *Streptococcus*, *Staphylococcus*, *Planococcaceae*, *Finegoldia*), and Proteobacteria (*Haemophilus*, *Burkholderia*) phyla. All samples broadly clustered into three microbiota profiles (Figure 2A). These were characterized by an enrichment in *Prevotella* genus for profile 1 (median relative abundance of 17%), *Lactobacillus* genus for profile 2 (median relative abundance of 37%), and a balanced representation of genera in profile 3. Alpha diversity analyses showed that microbiota profile 3 displayed the highest richness and diversity (Figure 2B,C). In addition, total bacterial load was evaluated using quantitative PCR with 16S targeted pan-bacterial primers. Interestingly, bacterial load was highest in the *Prevotella*-enriched samples (*p* < 0.05^∗^ and < 0.001^∗∗∗^ when compared to profiles 2 and 3, respectively) (Figure 2D).

**Table 1:** Detailed information about general information, spermiogram parameters and Bacterial 16S rRNA load in the normal and abnormal spermiogram groups.

	Normospermic group		Abnormal spermiogram group		
Number	26		68		
Age (years)	35.0 (23.2–46.0)		36.3 (25.0–61.0)		
Length of the couple infertility (years)	1.0 (0.5–8.0)		2.0 (0.5–16.0)		
Smoking (%)	22		11		

					**Patients below**
**Spermiogram parameters**	**median**	**range**	**median**	**range**	**the WHO limit**

Concentration (mio/ml)	50.5	(18.0–120.0)	15.0	(0–131.0)	34
Total count (mio)	151.6	(50.4–540.0)	50.0	(0–365.0)	30
Volume (ml)	3.0	(1.6–6.5)	3.3	(1.5–7.8)	0
Progressive motility (%)	47.0	(34.0–84.0)	29.0	(0–71.0)	40
Total motility (%)	70.5	(48.0–97.0)	45.0	(0–86.0)	28
Morphology (%)	4.0	(4.0–10.0)	2.0	(0–10.0)	63
Leukocyte count (10^6^/ml)	0	(0–1)	1	(0–10)	20

**Bacterial 16S rRNA (copies/ml)**	**n**		**n**		
< 10^3^	–		1		
10^3^–10^4^	6		6		
10^4^–10^5^	12		32		
10^5^–10^6^	5		19		
10^6^–10^7^	3		4		
10^7^–10^8^	–		6		
10^8^–10^9^	–		1		


### Semen Microbiota Community Structure

**FIGURE 3:**
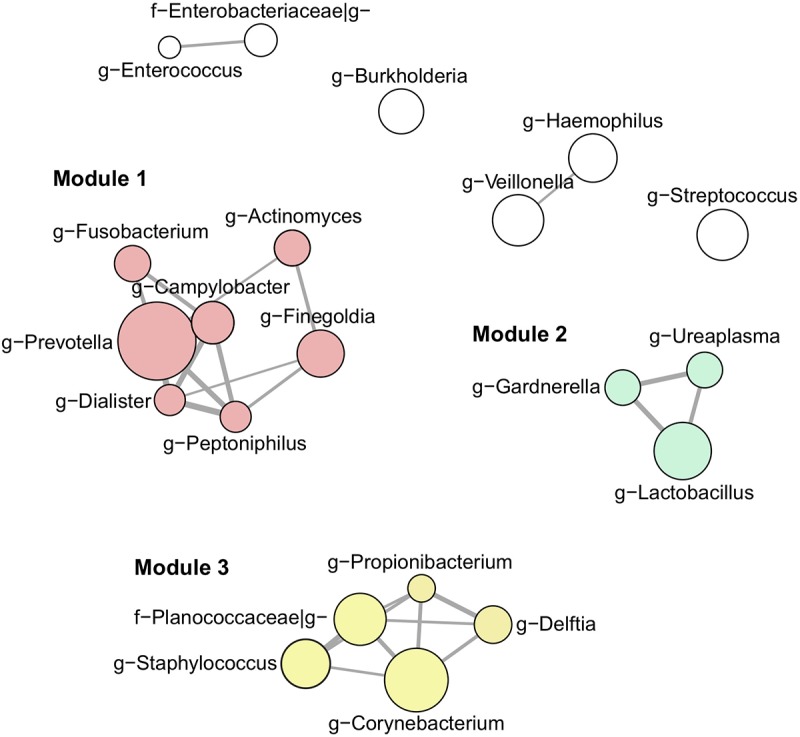
Interaction network of the most abundant genera (> 1% mean relative abundance) using SparCC algorithm. Connecting edges represent significant interactions (one-sided *p*-value < 0.05) with thickness proportional to SparCC co-occurrence values. Nodes are sized according to mean relative abundance of the corresponding genus in the data set. Members of interaction module 1, 2, and 3 are highlighted in red, green and yellow, respectively.

Our next aim was to investigate semen microbiota community structure and interactions using co-occurrence network analysis (Figure 3). Interaction network consisted of 21 nodes (most abundant bacterial genera, > 1% mean relative abundance) connected by 26 edges, as determined by the SparCC algorithm. These genera clustered into three main interaction modules, defined here as a group of a minimum 3 interconnected bacterial genera. Module 1 consisted of strictly anaerobic genera (*Prevotella, Finegoldia, Campylobacter, Actinomyces, Fusobacterium, Dialister, Peptoniphilus*), whereas module 2 contained facultative anaerobes (*Lactobacillus*, *Gardnerella*, *Ureaplasma*). Finally, module 3 included both strict and facultative anaerobes (*Staphylococcus*, *Corynebacterium*, *Propionibacterium*, *Planocaccaceae*, and *Delftia*).

### Impact of Specific Genera on Sperm Parameters

**FIGURE 4:**
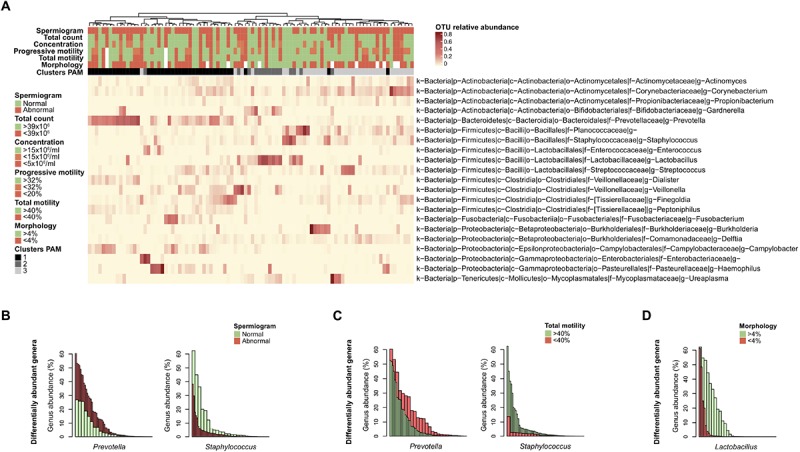
Differentially abundant bacterial genera with semen parameters. **(A)** Heatmap of the most abundant genera (> 1% mean relative abundance) with corresponding Ward linkage dendrogram based on the Bray Curtis distance matrix at genus level. Each column represents an individual sample and color-scale bar indicating operational taxonomic unit (OTU) relative abundance. Samples grouping according to spermiogram and microbiota profile (1-*Prevotella*-enriched, 2-*Lactobacillus*-enriched and 3-polymicrobial) are indicated as column annotations. OTUs are labeled based on their phylum (p), class (c), order (o), family (f), and genus (g). **(B)** Bar plot representation of differentially abundant bacterial genera as determined by linear discriminant analysis effect size (LEfSe) analysis comparing spermiogram groups. **(C)** Same analysis comparing total motility groups. **(D)** Same analysis comparing morphology groups. Each bar is colored according to its belonging to normal (green) or abnormal (red) group.

To evaluate the impact of the microbiota on sperm quality, patients were divided in normospermic and abnormal groups, based on spermiogram parameters (Figure 1). We did not observe any difference in alpha diversity measures (richness and diversity) between the two major phenotypes (Table 2A). This was also the case when stratifying patients according to specific spermiogram defects with the exception of a minor increase in the chao1 index in the group with an abnormal total motility parameter (Kruskal-Wallis or Chi-squared, *p* = 0.02^∗^) (Table 2A). We next used ANOSIM on the Bray Curtis distance matrix to compare microbiota composition among the two major phenotypes and the defect-specific subgroups (Table 2B). Overall microbiota composition did not differ between groups (Table 2 and Figure 4A).

**Table 2A:** Summary of alpha diversity analyses.

Kruskal-Wallis rank sum test among groups		

***Class***	***Kruskal-Wallis Chi-squared***	***P-value***
**Chao1 index (richness)**		
Spermiogram	1.046	0.306
Total count	0.0032237	0.955
Concentration	0.98128	0.612
Progressive motility	5.113	0.078
Total motility	5.4605	**0**.**021**
Morphology	1.7289	0.189
**Shannon index (diversity)**		
Spermiogram	1.8062	0.179
Total count	0.0079605	0.929
Concentration	4.1259	0.126
Progressive motility	3.3323	0.189
Total motility	2.7431	0.098
Morphology	1.7289	0.189


**Table 2B:** Summary of beta diversity analyses.

Analysis of similarity (ANOSIM) among groups		

***Class***	***R statistic***	***P-value***
Spermiogram	0.01193	0.359
Total count	-0.05727	0.967
Concentration	-0.05731	0.916
Progressive motility	-0.06103	0.962
Total motility	-0.03837	0.841
Morphology	0.02724	0.161


Finally, we used linear discriminant analysis effect size (LEfSe) to identify differentially abundant genera among the groups. *Prevotella* genus bacteria were significantly enriched in the group with abnormal spermiogram parameters (Figure 4B). On the other hand, *Staphylococcus* genus bacteria were significantly enriched in the normospermic group. To further dissect the relationship between discrete spermiogram parameters and the abundance specific genera, we performed a similar analysis using defect-specific subgroups. We found that the two-above mentioned genera were linked with differences in spermatozoa total motility (Figure 4C). Moreover, the relative abundance of the *Lactobacillus* genus was found to be enriched in samples with normal sperm morphology when compared to the corresponding control group (Figure 4D).

## Discussion

In this study, we explored the microbial content of the semen of men with normal and abnormal spermiogram parameters. Bacteriospermia was previously seen as a pathological condition and was associated with infertility, but several recent studies have shown that semen of fertile men harbors a unique microbiota (Hou et al., 2013; Liu et al., 2014; Weng et al., 2014; Zozaya et al., 2016). Given the limited number of studies, it is still unclear whether the presence of specific bacterial communities has the potential to influence sperm function. There is currently no clear consensus on the most appropriate hypervariable region of the 16S rRNA gene to sequence, each of those having their own advantages and limitations. Since two of the landmark seminal microbiota studies (Hou et al., 2013; Weng et al., 2014) have used V1-V2 regions for sequencing, we decided to follow the same strategy, which allowed us to directly compare our findings.

We identified three broad microbiota profiles with differences in richness, diversity and total bacterial load. Two of them were characterized by an enrichment of one particular genus, *Prevotella* and *Lactobacillus*, respectively. The third group did not have a predominant genus (polymicrobial). This is consistent with previous observations made in a Taiwanese study (Weng et al., 2014), which similarly identified three types of seminal microbiota communities, two of which match the ones presented here (*Lactobacillus*-predominant group and *Prevotella*-predominant group). In addition, Hou et al. (2013) also observed distinct seminal microbiota clusters with *Lactobacillus* and *Prevotella* being among the most represented genera in their study. The fact that three studies independently identified comparable microbiota profiles strongly supports the presence of highly conserved semen microbiota signatures among different world populations.

Using co-occurrence network analysis, we identified three main modules potentially reflecting microbial interactions in seminal fluids. Interestingly, module 1 and module 2 consisted of members previously identified as part of the commensal vaginal flora (*Prevotella, Lactobacillus, Finegoldia, Campylobacter, Actinomyces, Fusobacterium, Dialister, Peptoniphilus, Lactobacillus*, *Gardnerella*) (Ravel et al., 2011), while module 3 contained genera characteristic of the skin microbiota (*Corynebacterium*, *Staphylococcus*, *Planococcaceae*, *Propionibacterium*, *Delftia*) (Grice et al., 2009). The fact that modules 1 and 2 contained genera with similar oxygen requirements (strict anaerobes versus facultative anaerobes, respectively) suggests that the seminal fluid may offer different environmental milieus permissive to the survival of specific microbial populations with similar requirements. We did not observe any major difference in microbiota composition or diversity with semen parameters. LEfSe analysis, however, shed light on subtle changes in the relative abundance of specific bacterial genera. *Prevotella* genus was enriched in the abnormal group (at least one defective parameter) while *Staphylococcus* was associated with samples with normal spermiograms. This observation was held true when grouping the samples by total motility, indicating that this parameter may be the one mostly influenced by bacteria. Interestingly, *Prevotella*-enriched samples had the highest bacterial load and members of this genus are closely related with bacterial vaginosis in women (Zozaya-Hinchliffe et al., 2010; Srinivasan et al., 2013; Weng et al., 2014).

We also observed that samples with normal morphology were significantly enriched with *Lactobacillus* genus. Lactobacilli have been previously reported in normospermic samples and are known to positively influence the vaginal ecosystem (Younes et al., 2017). In addition, exposure of spermatozoa to lactobacilli has been shown to have a positive effect on motility and viability (Barbonetti et al., 2011). These observations are in agreement with previous culture-dependent studies, in which normospermic microflora was associated with the presence of Gram-positive bacteria (lactobacilli, coagulase-negative staphylococci, streptococci) (Ivanov et al., 2009; Mändar, 2013).

In summary, we observed that semen harbors unique microbiota profiles, which appear to be conserved across human populations. Many of the bacterial genera identified in this study were previously associated with vaginal microbiota. This is not surprising since microorganisms are exchanged during sexual intercourse (Mändar et al., 2015). In analogy with the vaginal counterpart, lower diversity of seminal microbiota seems linked with a healthy condition, which seems not to be the case for other body sites, including gut, lung or skin. Network analyses revealed that bacterial genera clustered in modules with similar oxygen requirements, arguing for different seminal microenvironments. One may hypothesize that microbiota could influence the milieu in which spermatozoa mature, thus impacting their physiology. Although we did not observe major differences in overall bacterial composition and diversity with sperm quality, we found that the differential abundance of specific bacterial genera, such as *Prevotella, Staphylococcus*, and *Lactobacillus* correlated with sperm motility and morphology deficiencies. Our results call for further studies of the bacterial colonization of the urogenital tract and, as with the female reproductive tract, opens potential niches for probiotic therapeutic avenues (Anahtar et al., 2018).

## Author Contributions

DB, NV, BM, and MS conceived the experimental design. DB and NV collected biological samples. DB, CP, VC, and MS processed the samples in the laboratory. CP processed and analyzed the sequencing data. DB, CP, NV, BM, and MS interpreted the data, prepared the figures and tables, and wrote the manuscript. All authors read and approved the final manuscript.

## Conflict of Interest Statement

The authors declare that the research was conducted in the absence of any commercial or financial relationships that could be construed as a potential conflict of interest.
