# Supplementary material for: Sperm Microbiota and Its Impact on Semen Parameters
Source: Front Microbiol. 2019 Feb 12;10:234. doi: 10.3389/fmicb.2019.00234 (PMC6379293; doi:10.3389/fmicb.2019.00234)
Supplement: Supplementary file 1 [file Table_1.DOCX]

Supplementary table 1 – Classification of spermiogram parameters of each sample included in the study. Asterisks denote samples with at least one spermiogram parameter below the WHO reference values.

| **ID** | **Concentration (x10^6^/ml)** | **Total count (x10^6^)** | **Progressive motility (%)** | **Total motility (%)** | **Morphology (%)** |
| --- | --- | --- | --- | --- | --- |
| Sperm4* | more15 | more39 | more32 | more40 | less4 |
| Sperm7* | less5 | less39 | less20 | less40 | less4 |
| Sperm8 | more15 | more39 | more32 | more40 | more4 |
| Sperm14* | less15 | less39 | less20 | less40 | less4 |
| Sperm17* | less15 | less39 | more32 | more40 | less4 |
| Sperm21 | more15 | more39 | more32 | more40 | more4 |
| Sperm25* | more15 | more39 | more32 | more40 | less4 |
| Sperm26* | more15 | more39 | less32 | less40 | less4 |
| Sperm36* | more15 | more39 | less20 | less40 | less4 |
| Sperm40* | less5 | less39 | more32 | less40 | less4 |
| Sperm45 | more15 | more39 | more32 | more40 | more4 |
| Sperm47* | less5 | less39 | less20 | less40 | less4 |
| Sperm48* | more15 | more39 | less20 | less40 | less4 |
| Sperm52* | more15 | more39 | more32 | more40 | less4 |
| Sperm54* | less15 | more39 | less32 | more40 | less4 |
| Sperm58 | more15 | more39 | more32 | more40 | more4 |
| Sperm60* | less5 | less39 | less20 | less40 | less4 |
| Sperm69* | more15 | more39 | less32 | more40 | less4 |
| Sperm70* | more15 | more39 | more32 | more40 | less4 |
| Sperm77* | less15 | less39 | less32 | more40 | less4 |
| Sperm78* | more15 | more39 | more32 | more40 | less4 |
| Sperm80* | more15 | more39 | more32 | more40 | less4 |
| Sperm81* | less5 | less39 | less32 | less40 | less4 |
| Sperm82* | less15 | less39 | more32 | more40 | less4 |
| Sperm83* | less5 | less39 | more32 | more40 | less4 |
| Sperm84* | more15 | more39 | more32 | more40 | less4 |
| Sperm85* | less5 | less39 | less32 | more40 | less4 |
| Sperm87 | more15 | more39 | more32 | more40 | more4 |
| Sperm88* | less5 | less39 | more32 | more40 | less4 |
| Sperm89* | less5 | less39 | less20 | less40 | less4 |
| Sperm90* | more15 | more39 | less32 | less40 | less4 |
| Sperm91* | more15 | more39 | more32 | more40 | less4 |
| Sperm94 | more15 | more39 | more32 | more40 | more4 |
| Sperm96* | more15 | more39 | less32 | less40 | less4 |
| Sperm97 | more15 | more39 | more32 | more40 | more4 |
| Sperm98* | less15 | less39 | less20 | less40 | less4 |
| Sperm99 | more15 | more39 | more32 | more40 | more4 |
| Sperm100 | more15 | more39 | more32 | more40 | more4 |
| Sperm101* | less5 | less39 | more32 | more40 | less4 |
| Sperm104* | less15 | less39 | less20 | less40 | more4 |
| Sperm105* | more15 | more39 | less32 | less40 | less4 |
| Sperm108* | more15 | more39 | more32 | more40 | less4 |
| Sperm109 | more15 | more39 | more32 | more40 | more4 |
| Sperm110 | more15 | more39 | more32 | more40 | more4 |
| Sperm111* | more15 | more39 | less32 | more40 | less4 |
| Sperm112 | more15 | more39 | more32 | more40 | more4 |
| Sperm113 | more15 | more39 | more32 | more40 | more4 |
| Sperm114* | less5 | less39 | less20 | less40 | less4 |
| Sperm115 | more15 | more39 | more32 | more40 | more4 |
| Sperm116* | more15 | less39 | more32 | more40 | less4 |
| Sperm117* | less5 | less39 | less32 | less40 | less4 |
| Sperm118* | less5 | less39 | less20 | less40 | less4 |
| Sperm119* | less15 | less39 | less32 | less40 | less4 |

Supplementary table 1 – Cont.

| **ID** | **Concentration (x10^6^/ml)** | **Total count (x10^6^)** | **Progressive motility (%)** | **Total motility (%)** | **Morphology (%)** |
| --- | --- | --- | --- | --- | --- |
| Sperm120* | more15 | more39 | less32 | more40 | less4 |
| Sperm121 | more15 | more39 | more32 | more40 | more4 |
| Sperm122 | more15 | more39 | more32 | more40 | more4 |
| Sperm123* | less15 | more39 | more32 | more40 | less4 |
| Sperm127* | more15 | more39 | less32 | more40 | more4 |
| Sperm128* | more15 | more39 | less32 | more40 | less4 |
| Sperm129* | less15 | more39 | more32 | more40 | less4 |
| Sperm130* | more15 | more39 | less32 | less40 | less4 |
| Sperm131 | more15 | more39 | more32 | more40 | more4 |
| Sperm132* | less5 | less39 | less20 | less40 | less4 |
| Sperm133 | more15 | more39 | more32 | more40 | more4 |
| Sperm134* | more15 | more39 | more32 | more40 | less4 |
| Sperm135 | more15 | more39 | more32 | more40 | more4 |
| Sperm136* | more15 | more39 | less32 | more40 | less4 |
| Sperm137 | more15 | more39 | more32 | more40 | more4 |
| Sperm139* | less15 | less39 | less32 | less40 | less4 |
| Sperm140* | less15 | more39 | less20 | less40 | less4 |
| Sperm143 | more15 | more39 | more32 | more40 | more4 |
| Sperm144* | more15 | more39 | more32 | more40 | less4 |
| Sperm145 | more15 | more39 | more32 | more40 | more4 |
| Sperm147* | more15 | more39 | less32 | more40 | more4 |
| Sperm148* | more15 | more39 | less32 | more40 | less4 |
| Sperm150* | less5 | less39 | less20 | less40 | less4 |
| Sperm151* | less5 | less39 | less20 | less40 | less4 |
| Sperm152 | more15 | more39 | more32 | more40 | more4 |
| Sperm153* | more15 | more39 | less32 | less40 | less4 |
| Sperm154* | less15 | less39 | less32 | less40 | less4 |
| Sperm155* | less15 | more39 | more32 | more40 | less4 |
| Sperm156* | less15 | less39 | less32 | more40 | less4 |
| Sperm157* | more15 | more39 | more32 | more40 | less4 |
| Sperm160 | more15 | more39 | more32 | more40 | more4 |
| Sperm162* | more15 | more39 | more32 | more40 | less4 |
| Sperm164* | less15 | less39 | more32 | more40 | more4 |
| Sperm165* | less5 | less39 | less20 | less40 | less4 |
| Sperm166* | less15 | less39 | more32 | more40 | more4 |
| Sperm168 | more15 | more39 | more32 | more40 | more4 |
| Sperm169* | more15 | more39 | more32 | more40 | less4 |
| Sperm170 | more15 | more39 | more32 | more40 | more4 |
| Sperm174* | more15 | more39 | less32 | more40 | less4 |
| Sperm175* | more15 | more39 | more32 | more40 | less4 |
| Sperm183* | more15 | more39 | more32 | more40 | less4 |
